# Towards cleaner environment: recycling microalgal co-product to reduce emissions and impacts while eliminating fishmeal in rainbow trout feed for sustainable aquaculture

**DOI:** 10.1007/s11356-024-34136-6

**Published:** 2024-07-09

**Authors:** Pallab K. Sarker, Ebenezer Figueroa, Anne R. Kapuscinski, Brandi McKuin, Benjamin V. Schoffstall, Devin Fitzgerald, Connor Greenwood, Kira O’Shelski, Emily Noelle Pasion, Duncan Gwynne, Diego Gonzalez Orcajo, Sofie Andrade, Pablo Nocera

**Affiliations:** 1 Environmental Studies Department, University of California Santa Cruz, Santa Cruz, CA 95060 USA; 2 Earth and Oceanographic Science, Bowdoin College, Brunswick, ME 04011 USA; 3 School of Engineering, University of California Merced, 5200 North Lake Rd, Merced, CA 95343 USA; 4 Department of Microbiology and Environmental Toxicology, University of California Santa Cruz, Santa Cruz, CA 95060 USA

**Keywords:** Fishmeal, Aquaculture feed, Defatted microalgal biomass, Sustainability, Rainbow trout, Eutrophication, Environmental impact

## Abstract

The rapid increase in aquaculture over the last several decades has led to concerns about the environmental impact of fish feeds relying on marine resources for fishmeal (FM). We aim to assess *Nannochloropsis* sp. QH25 co-product as a viable and sustainable replacement for FM in juvenile rainbow trout, *Oncorhynchus mykiss*, feeds. We formulated four experimental diets: a reference (FM based), 33N, 66N, and 100N diet (33%, 66%, and 100% co-product replacement). Rainbow trout were randomly assigned to one of 16 tanks and randomly assigned an experimental diet to consume throughout the experiment (64 days total), with four replicate tanks per diet. We compared the phosphorus (P) and nitrogen (N) digestibility, emissions, and growth between diets and, compared six environmental impacts (biotic resource use (BRU), global warming potential (GWP), water use, land use, marine eutrophication potential (MEP), and freshwater eutrophication potential (FEP)) of each diet. Our results indicate that replacing FM with co-product did not significantly alter growth. P digestibility of the experimental and reference diets was comparable. BRU conversion ratio was significantly lower in the experimental diets. However, there were significantly higher water and land use conversion ratios but insignificantly higher results in GWP, MEP, and FEP between the reference and 100N diet.

## Introduction

Although the rapid increase in aquaculture over the last 40 years has led to a reduction of overexploitation of wild fish (Diana 2009; Sarker et al. 2020b, a; Fisheries Division, Food and Agriculture Organization of the United Nations (FAO), Rome, Italy and Miao 2020; Naylor et al. 2021) and claims of increasing food security across the globe (Béné et al. 2016; FAO 2022), the environmental impact of this growing industry is continuously brought into question (Naylor et al. 2021). While aquaculture has helped reduce the overexploitation of fish, capture fisheries is still the dominant method for production, especially for marine capture fisheries (FAO 2022). The aquaculture industry is a primary user of fish-derived products which is associated with environmental concerns that extend beyond overexploitation of wildlife (Ghamkhar and Hicks 2020; FAO 2022; Andrade et al. 2024).

One of the leading causes of concern in the aquaculture industry is phosphorus (P) emissions which can lead to eutrophication of fresh and marine water ecosystems, among other environmental impacts (Olsen et al. 2008; Sarker et al. 2011; Huang et al. 2020; Gamble et al. 2021). Therefore, it is essential to reduce the P from aquaculture systems, which can be done through the manipulation of fish feeds.

Current aquaculture feeds rely on fishmeal (FM) fish oil (FO) and terrestrial crops. FM often contains indigestible forms of P, such as hydroxyapatite, (Baruah et al. 2004; Sarker et al. 2011) and is sourced from marine resources (Naylor et al. 2009) which increases P loading into aquaculture systems, extraction of limited marine resources, and environmental impacts (Naylor et al. 2021; McKuin et al. 2023). Terrestrial crops have been included into fish feed formulations to relieve the over extraction of wild fish resources. However, reducing reliance of FM in aquafeeds is especially a challenge for salmonids, which require high amounts of protein, amino acids, and fatty acids to meet growth performance and overall health requirements (Oliva-Teles et al. 2015; Sarker et al. 2020b). Terrestrial crops, such as soy and corn, contain hexakisphosphate (phytate P), an indigestible P to fish, and is a major contributor to P emissions resulting from aquafeeds (Baruah et al. 2004; Herath and Satoh 2015; Gamble et al. 2021). Fish do not have the enzyme to process phytate P, which suppresses nutrient digestibility, resulting in large amounts of undigested P excreted by the fish, leading to eutrophication (Baruah et al. 2004; Kokou and Fountoulaki 2018). Furthermore, the use of terrestrial crops to replace FM has many disadvantages such as lack of essential amino acids for fish growth, low digestibility, and high life cycle environmental impact including high P emissions (Li et al. 2009; Pereira et al. 2012; Sarker et al. 2013; Sarker 2014; Gephart et al. 2017; McKuin et al. 2023).

One promising alternative to FM and terrestrial crops with potential for higher P retention and lower environmental impacts is marine microalgae. Current industrial practices underutilize protein rich, defatted marine microalgal co-product making it a potentially more sustainable alternative to conventional fish feeds (Khan et al. 2018; Sarker et al. 2020b). Marine microalgae shows potential for salmonids, such as rainbow trout, due to its nutritional value and impact on growth (Ahmed et al. 2014; Shah et al. 2018; Trevi et al. 2023; Sarker 2023).

This study examines how the microalgal co-product *Nannochloropsis* sp. QH25, as a replacement for FM, can be utilized to decrease P and emissions and lower other environmental impact of aquafeeds for juvenile rainbow trout, a salmonid essential to aquaculture (Sarker et al. 2020b). We focused the research presented here on evaluating the novel alternative microalgal co-product, by rainbow trout as an important model for all farmed salmonids. Because Atlantic salmon (*Salmo salar*) and rainbow trout (*Oncorhynchus mykiss*) aquaculture used approximately 24–30% of FM and 50–60% of FO destined for aquafeeds (Shepherd and Jackson 2013; Willer et al. 2022). Also, due to the high demand for salmonids like rainbow trout (FAO 2022), finding sustainable alternatives to FM that meet the nutrient requirements of the carnivorous fish is a challenge to the aquaculture industry (Sarker et al. 2020b; Ghamkhar and Hicks 2021; Bruni et al. 2021; Melenchón et al. 2022). The aquafeed industry is seeking good protein ingredients that can replace FM use in aquaculture diet. This study focuses on *Nannochloropsis* sp. QH25 co-product because the microalgae industry seeks marketable uses of the large volumes of this microalgal co-product protein-rich biomass left-over after oil extraction for valuable products such as nutraceuticals. Alternative microalgal co-product could be potential to fill the raw material gap. First, we assessed P loading of experimental diets containing *Nannochloropsis* sp. QH25 at varying levels of FM replacement over a given study period and compared it to the P loading of a reference diet. Secondly, we identified the environmental tradeoffs of different FM replacement levels of the diet. We used the Cruz Aquafeed Sustainability Tool (CAST) to estimate the environmental impact conversion ratios (defined as the product of the environmental impact of the compound feed and the feed conversion ratio) of the experimental diets including the environmental impact categories (biotic resource use (BRU), global warming potential (GWP), water use, land use, marine eutrophication potential (MEP), and freshwater eutrophication potential (FEP) and tradeoffs to different FM replacement levels.

To our knowledge, there are no other publications which report nutrient budgets, environmental impacts, and economic conversion ratios when *Nannochloropsis* sp. QH25 is used as a sustainable FM replacement for rainbow trout species (McKuin et al. 2023; Sarker et al. 2020b).

Our objectives for this study include the following:

1. Developing phosphorous and nitrogen budgets to assess how the inclusion of *Nannochloropsis* sp. QH25 co-product in rainbow trout aquafeeds impact nutrient loading
2. Estimate environmental impacts and economic conversion ratio of utilizing *Nannochloropsis* sp. QH25 as a novel rainbow trout aquafeed ingredient at various inclusion levels.

## Materials and methods

### Growth experiment dietary design

To meet the optimal growth requirements of juvenile rainbow trout (Table 1), we formulated four iso-nitrogenous, iso-energetic, and iso-lipidic experi­mental diets (Sarker et al. 2020b). A reference feed was formulated based on a current commercial feed containing 7.5% FM, 14% FO, and 0% *Nannochloropsis* sp. QH25 co-product. Three fish-free experimental diets used *Nannochloropsis* sp. QH25 co-product to replace 33% (33N), 66% (66N), and 100% (100N) of FM relative to the reference diet. All diets only differed in the amount of FM and *Nannochloropsis* sp. QH25 co-product used, with *Nannochloropsis* sp. QH25 comprising 0%, 4.1%, 7.4%, and 10.0% of the diet by weight (reference, 33N, 66N, 100N experimental diets, respectively). Recently, we conducted a growth experiment by combining whole cells of marine in rainbow trout to replace both FM and FO via fish-free feed formulations exhibited a minor, but significant, lower growth (Sarker et al. 2020b). Then, we decided to conduct step-wise experiments including a feeding trial that compared serial replacement of fishmeal. Thus, in this study, we formulated feeds and conduct a nutritional feeding experiment to identify a suitable level of fishmeal replacement by *Nannochloropsis* sp. QH25 co-product meal. We formulate four iso-nitrogenous, iso-energetic, and iso-lipidic experimental diets, in which processed co-product meal replaces 0, 33, 66, and 100% of the fish meal. The nutrient-rich *Nannochloropsis* sp. QH25 co-product used in this experiment was supplied by Qualitas Health Inc., an industry leader in sustainable microalgae cultivation that markets EPA-rich oil extracted from *Nannochloropsis* sp. QH25 (Kagan and Matulka 2015; iwi life iwi life 2024).

**Table 1:** Formulation: reference diet without *Nannochloropsis* sp. QH25 co-product meal, and three diets with different replacement levels of FM by raw co-product meal

Ingredient (%)	Diet			
	Reference	33N	66N	100N
Fish meal	7.5	5.02	2.55	0
Fish oil	14	14	14	14
Co-product meal (raw)	0	4.1	7.4	10
Feather meal	15	15	15	15
Blood meal	7	7	7	7
Corn gluten meal	20	20	20	20
Soy protein concentrate	20	20	20	20
Wheat gluten	5	5	5	5
CaHPO4	1	1	1	1
Vitamin-mineral premix	0.6	0.6	0.6	0.6
Lysine	1	1	1	1
Methionine	0.2	0.2	0.2	0.2
Choline chloride	0.5	0.5	0.5	0.5
Alpha cellulose	4.5	3.07	2	2
Ascorbic acid	0.2	0.2	0.2	0.2
Taurine	0.5	0.5	0.5	0.5
Lecithin	3	3	3	3
Astaxanthin	0.05	0.05	0.05	0.05

Each diet contained yttrium oxide (Y2O3), an indigestible marker sourced from Thermo Scientific, Waltham MA, USA, in the basal diet at a rate of 1.0%. To create the feed, we first mixed all micro-ingredients followed by macro-ingredients which were slowly added and thoroughly mixed into the feed to maintain a homogenous texture. Diets were manufactured at the Kapuscinski-Sarker Lab space in Natural Sciences II (University of California, Santa Cruz CA, USA) using a single-screw extruder (TT-100 tabletop lab scale extruder from Akron Tool and Die, Akron Ohio, USA). During extrusion, the diet was exposed to an average target temperature in the barrels at 90 °C and passed through the extruder for 18 s exposure. All diets were top coated with FO using a rotating mixer (SUNCOO 4/5HP Electric Concrete Cement Mixer 5 Cu Ft Mortar Mixing Stucco Seeds Portable Barrow Machine) and 24-mm mercury pressure. Mixing was carried out for 15 min. After mixing, the feed was dried overnight to below 10% moisture content in a drying cabinet. The pellets were then sieved and stored at − 20 °C. Pellets used at the initial stages of the experiment were 2.0 mm in size. As the fish aged, the pellet size was increased to 4.0 mm to meet the nutritional demands of the larger fish.

### Fish husbandry, feeding, and feces collection

Prior to the digestibility trial, we randomly allocated juvenile rainbow trout with an average weight of 40.46 ± 1.44 g in 200-gal rectangular tanks (30 fish/tank, four tanks/dietary treatment, total trout for 16 tanks) of freshwater recirculating aquaculture systems (RAS) at the University of California, Santa Cruz CA, USA. Following the placement into the tanks, the rainbow trout were fed the reference diet for a 7-day acclimation period. After the acclimation period, fish were randomly assigned an experimental diet to forage on until apparent satiation twice a day, in the morning and afternoon, 6 days a week, for 64 days (Sarker et al. 2020b). A total of 16 tanks were used with four replicate tanks per diet.

Each tank was monitored to maintain recommended conditions for rainbow trout. Dissolved oxygen, dissolved oxygen saturation, temperature, and pH were sampled daily using a handheld YSI 1020Pro multiparameter meter to keep dissolved oxygen at 8.7 mg/L, water temperature at 15.4 °C, and pH at 8.6. Ammonia, nitrite, nitrate, and alkalinity of the water were sampled weekly using a benchtop YSI 9500 spectrophotometer to maintain total ammonia nitrogen at 0.2 mg/L, nitrite nitrogen at 0.1 mg/L, and nitrate nitrogen 26.8 at mg/L.

Fish fecal samples were collected before feeding daily using a radial flow settler. The radial flow settler was installed between the culture tank outflow and sump tank inflow to collect intact fecal matter at the bottom of the system. To prevent contamination, uneaten feed pellets were siphoned out of the radial flow settler. Intact solid fecal matter was gently removed from a separate collection bin using pipettes. We placed the fecal matter in a 50-mL Falcon tube (BD Falcon™) where it was allowed to settle at the bottom of the tube. Once fecal samples were settled, we removed supernatant water at the top using a pipette. Then, fecal samples were frozen at − 20 °C. We pooled fecal samples from every collection from each specific tank during the experiment. At the end of the experiment, we lyophilized, finely ground, and stored samples at − 20 °C for P analysis.

To determine P retention, prior to the start of the experiment, five rainbow trout were euthanized, homogeneously ground, freeze-dried for 48 h, reground, and stored at a constant temperature of − 20 °C until the P content was analyzed (Gamble et al. 2021). We repeated this process on three fish from each tank on the final sampling day of the experiment.

Prior to P analysis, feces from each tank were collected, frozen, dried, and homogenized. To determine P content, feces samples and three whole body fish samples per tank were collected and analyzed. Prior to analysis, the fecal collector of each tank was emptied out. Feces were then separated from non-fecal material, freeze dried, and stored until analysis. Only tanks with fecal samples of at least 0.1 ± 0.002 g were used for analysis, totaling 13 fecal matter samples P analysis.

Phosphorus content of three samples of each diet were analyzed. Mean P content for each diet was used to calculate the P budget and digestion analysis (Gamble et al. 2021).

### Biological sampling procedures, filet preparations, and growth measurements

We bulk-weighed the fish at the beginning of the experiment and repeated this process every 3 weeks until the end of the experiment (64 days). We did not feed fish 24 h before each bulk-weight sampling. During final sampling, the entire fish biomass of every tank was weighed. Three fish per tank were fileted for whole-body proximate analysis from a standardized dorso-anterior landmark, packaged in sterile polythene bags (Whirl–pak, Naso, Fort Atkinson, Wisconsin), and stored frozen (− 20 °C) until fatty acid analysis. We determined the dietary effects on growth by evaluat­ing final weight and feed conversion ratio (FCR). We calculated the indices as follows (Sarker et al. 2020a):

1 $$FCR=feed\;intake/weight\;gain$$

### Nutrient digestibility analysis and calculations

For P analysis, diets and fecal matter were mixed to homogeneity separately. We added 10 mL of nitric acid, HNO₃, to the homogenized diet and fecal matter samples. Each sample was refluxed for 10 min. Then, 5 mL of concentrated nitric acid was added and refluxed for 30 min. We added 2 mL of water and 3 mL of hydrogen peroxide, H₂O₂, and continued to add 1 mL aliquots of H₂O₂ until bubbling of samples subsided. Once the bubbling subsided, we added 10 mL of concentrated HCl to the samples and refluxed for an additional 15 min. All analysis was conducted at Kapuscinski-Sarker lab at Natural Science 2, Environmental Studies Dept, University of California, Santa Cruz.

We prepared all samples for ICP OES (Thermo iCap 7400 radial view ICP-OES, Optical Emission Spectroscopy conducted at UC Santa Cruz Plasma Analytical Laboratory, RRID:SCR_021925) to analyze elemental composition (phosphorus and yttrium oxide) of diets and feces. The absorbance values from this sampling allowed us to obtain the P concentration of the diets. Yttrium oxide was used to measure how much digestible P was present in the feed and feces.

The apparent digestibility coefficient (ADC) for the four diets was calculated using the equation (Cho et al. 1982):

2 $$ADC=100\times \left[1-\left(\frac{\%Nutrient \;of\; feces}{\%Nutrient \;of \;diet}\right)\times \left(\frac{\%Marker \;in\; diet}{\%Marker\; in\; feces}\right)\right]$$

To calculate the % of digestible Nutrient (g/kg) of feed, the ADC was multiplied by the amount of nutrient in each diet. The nutrient budget for each diet was calculated from (Cho et al. 1982; Gamble et al. 2021). To identify the nutrient intake per gram of fish, we multiplied the feed per fish dry weight by the percent of nutrient present in the dry feed as follows:

3 $$Intake\;Nutrient\left(g/fish\right)=\left(\left(\frac{feed}{fish\;dry\;weight}\right)\times \left(feed\;Nutrient\;dry\%\right)\right)/100$$

To get the nutrient intake, g per kg, we took the intake nutrient (g/fish) and divided it by the difference in final and initial dry weight of fish as follows:

4 $$Intake\;of\;nutrient\left(g/kg\right)=\frac{Intake \;nutrient\left(g/fish\right)}{ Final \;dry\frac{weight}{fish\left(g\right)}-Initial \;dry weight/fish\left(g\right)}\times 100$$

To calculate nutrient retention, we did the following;

5 $$Retained\; Nutrient/gfish=\frac{\left(final\; dry\frac{weight}{fish\left(g\right)}\times final \;fish\; Nutrient\%\right)}{100}\times \frac{\left(initial \;dry\frac{weight}{fish\left(g\right)}\times initial\; fish \;Nutrient\%\right)}{100}$$

Followed by:

6 $$Retained\; Nutrient\left(g/kg\right)= \frac{retained\; Nutrient/gfish}{Final \;dry\frac{weight}{fish\left(g\right)}-initial\; dry \;weight/fish\left(g\right)}\times 1000$$

To assess nutrient loading, we calculated the solid and dissolved nutrient waste from the fish. We calculated solid nutrient loading on g/kg feed using the equations:

7 $$Solid\; Nutrient\left(g/fish\right)=Intake\; Nutrient\left(g/fish\right)\times \left(1-\left(Diet \;ADC\; of \;Nutrient\%/100\right)\right)$$

8 $$Solid \;Nutrient\left(g/kg\right)=\frac{Solid\; Nutrient\left(g/fish\right)}{Final\; dry\frac{weight}{fish\left(g\right)}-initial\; dry weight/fish\left(g\right)}\times 1000$$

Then, we would use the solid nutrient (g/kg) to calculate the dissolved nutrient loading using the following equations:

9 $$Rejected \;Nutrient\left(g/kg\right)=Intake \;Nutrient\left(g/kg\right)-Retained\; Nutrient\left(g/kg\right)$$

10 $$Dissolved\; Nutrient\left(g/kg\right)=Rejected \;Nutrient\left(g/kg\right)-solid\; Nutrient\left(g/kg\right)$$

### Environmental impact conversion ratio

We used the calculator module in CAST (McKuin et al. 2024; available at https://cast.sites.ucsc.edu/) to estimate the environmental impact conversion ratio (kg emission per kg of fish) of the reference and experimental diets (33N, 66N, and 100N feeds). The environmental impact conversion ratio is defined as the product of the environmental impacts (kg emission per kg compound feed) and the feed conversion ratios (kg compound feed per kg fish). Instead of using the default feed conversion ratios computed by CAST, we input the feed conversion ratios of the reference and experimental diets.

In CAST, the environmental impacts are calculated using life cycle inventory data (including the cultivation, harvesting, and processing stages of the ingredients) and environmental impact characterization factors. The life cycle inventory data for *N.* sp. QH25 was sourced from McKuin et al. (2023). FM and FO were sourced from McKuin et al. (2022), feather meal was sourced from McKuin et al. (2023), and the other ingredients (corn gluten meal, blood meal, soy protein concentrate, and wheat gluten meal) were sourced from the Agri-footprint database (v. 2.0) (Blonkconsultant 2015). For *Nannochloropsis* sp. QH25, FM, FO, and feather meal, the Ecoinvent 3.4 database was used for background data (e.g., electricity, water supply, heat generation, and crop ingredients; (Wernet et al. 2016)). For all ingredients, CAST uses The ReCiPe 2016 Midpoint (H) v.1.02 method (Huijbregts et al. 2017) characterization factors to calculate the GWP, water consumption, land use, FEP, and MEP categories. For BRU estimates, CAST uses values from the literature and LCA databases (see McKuin et al. 2023).

The CAST’s calculator module works by multiplying a user’s inputs of the feed conversion ratio of rainbow trout diet, the individual feed ingredient amounts in the rainbow trout diet, and each of the entered ingredient’s corresponding environmental impact value sourced from the stored life cycle analysis database for each environmental impact. This is further explained with the following equation:

11 $${EICR}_{i,k}=\sum_{j}^{N}{CF}_{j}\bullet {EI}_{j,k}\bullet {FCR}_{i,j}$$

where *EICR* is the environmental impact conversion ratio of the rainbow trout (environmental impact kg fish^−1^), *CF* is the compound feed ingredient (kg feed), *FCR* is the feed conversion ratio which is a conventional measure of aquaculture and livestock production efficiency: the weight of feed intake divided by weight gained by the fish (kg compound feed kg fish^−1^), *i* is the rainbow trout species and *j* is the ingredient, *EI* is the environmental impact of the selected ingredient (EI kg ingredient^−1^), and *k* is the environmental impact metric (global warming potential in units kg CO_2_e, water use in units m^3^ water use, land use in units m^2^ land use, marine eutrophication potential in kg N, freshwater eutrophication potential in units P, and biotic resource use in units kg C).

### Statistical analysis

A one-way ANOVA was run to analyze the variance of P (g/kg), ADC of P, P digested, and P budget (intake, retention, solid, and dissolved) of each diet. Standard error was calculated, and Tukey’s test of multiple comparisons were run to identify statistical differences where *p* values were below 0.05. Environmental impact figures were generated using the sum of the environmental impact conversion ratios of each ingredient for every diet. We then multiplied the feed conversion ratio by the total environmental impact conversion ratio for each replicate tank. These values were then used to find the standard error for the diets. Tukey’s tests of multiple comparisons were also run for each environmental impact to identify similarities and differences between diets where *p* values were less than or equal to 0.05.

## Results and discussion

### Growth

**Fig. 1:**
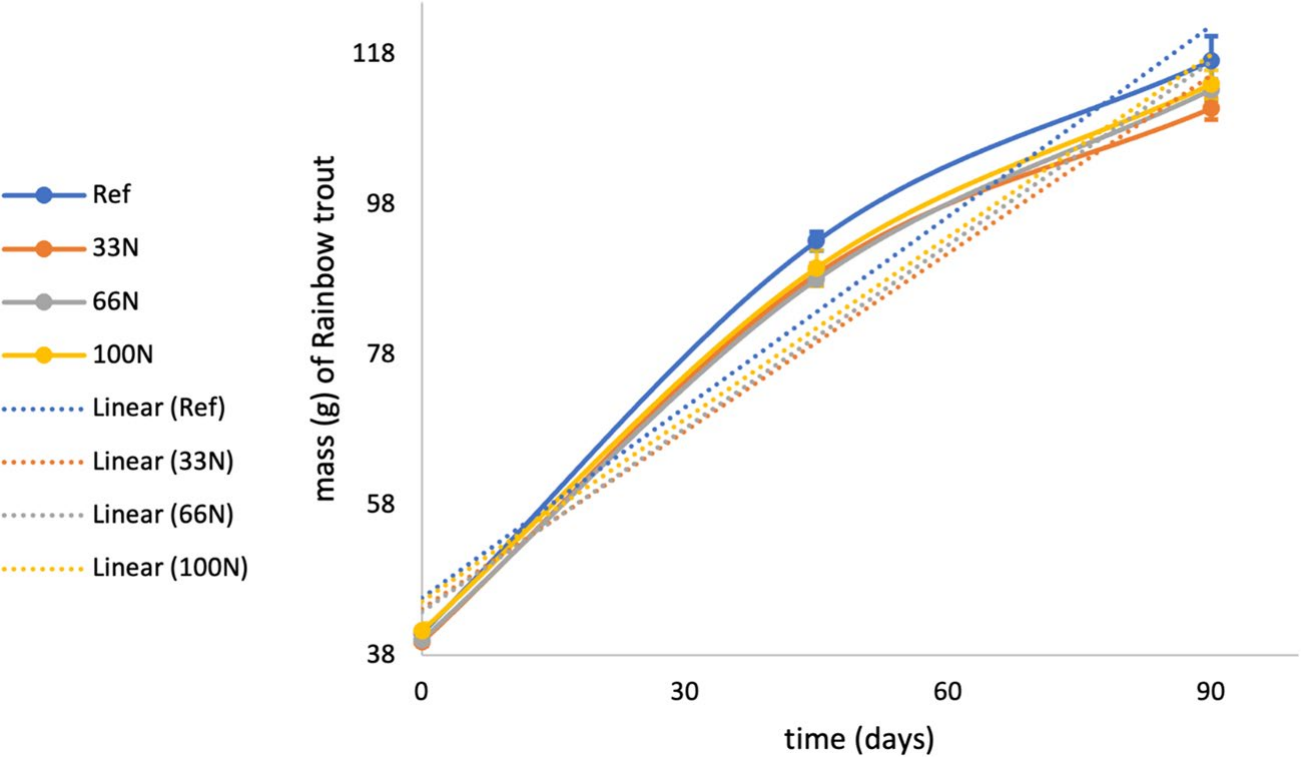
Growth curves for each treatment from day 0 to day 90 for four different diets: no replacement of FM (Ref), replacement of 33% of FM with *Nannochloropsis* sp. QH25*.* (33N), replacement of 66% of FM with *Nannochloropsis* sp. QH25 (66N), and replacement of 100% of FM with *Nannochloropsis* sp. QH25 (100N). Linear regression equations and coefficient of determination (*R*^2^) values: Ref, *y* = 0.847*x* + 45.635 (*R*2 = 0.9564); 33N, *y* = 0.7898*x* + 44.185 (*R*2 = 0.9543); 66N, *y* = 0.8144*x* + 43.799 (*R*2 = 0.9687); and 100N, *y* = 0.8086*x* + 45.194 (*R*2 = ^0.9653). Error bars show standard error of the mean (^*n* = 4 replicates per diet)

While the fish growth of the reference diet appears to be higher over time, fish growth was not statistically different (*p* > 0.05) between the reference and 100 N diet by the end of the experiment (Fig. 1). Furthermore, growth across all diets, reference, 33N, 66N, and 100N, were not statistically different from each other (*p* > 0.05). All diets followed similar trends with similar linear regressions and high correlation coefficients between diets and sampling points.

The results from this study indicate that replacing FM using the microalga *Nannochloropsis* sp*.* QH25 does not significantly alter the growth of rainbow trout. Previous studies suggest that higher inclusions of microalgae co-product, specifically *N. oculata*, in tilapia and other lower trophic level fish feeds yielded better growth results (Sarker et al. 2018). However, previous studies also indicate that *Nannochloropsis* sp. QH25 can be included in higher trophic level fish in moderation. Sørensen et al. (2017) found that *Nannochloropsis* sp. could be an alternative to FM in Atlantic salmon feeds if inclusion was modest. *Nannochloropsis* sp. QH25 inclusions at 10% did not negatively affect growth performance of the Atlantic salmon. Higher inclusions of *Nannochloropsis* sp. QH25 yielded less weight gain and lower specific growth rates in salmon (Sørensen et al. 2017; Liu et al. 2022). While this is a potential challenge to the incorporation of *Nannochloropsis* sp. QH25 to higher trophic fish diets, Gong et al. (2020) found that preprocessing of *Nannochloropsis* sp. QH25 via extrusion would make nutrients more accessible, and therefore improve growth of the fish. Though not significantly different, our results showed the highest growth in the reference diet, followed by the 100N, 66N, and 33N, respectively. Although our results agree with the hypothesis that higher inclusions of microalgal co-product lead to comparable growth results (Sarker et al. 2018), they challenge what other studies say for fish growth in higher trophic levels.

### P digestibility and loading

**Fig. 2:**
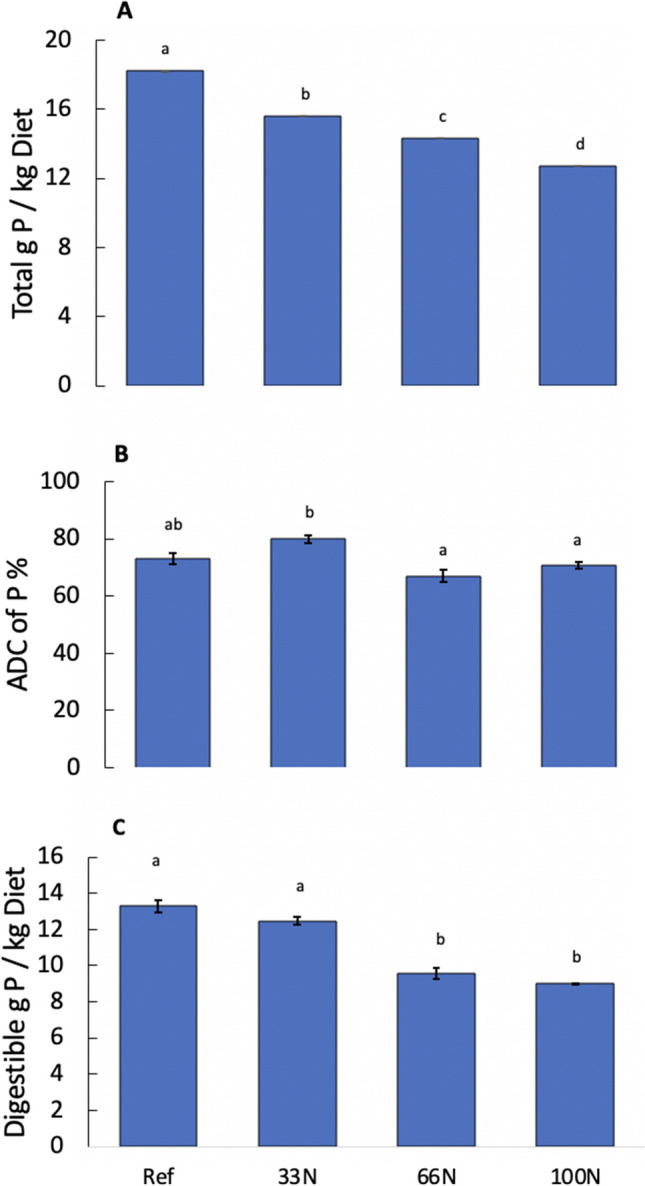
**A** Average quantity of phosphorus in reference and experimental diets. Experimental diets contained *Nannochloropsis* sp. QH25 that replaced 33N, 66N, or 100N of FM in the feeds (*n* = 4 replicates for reference and 33N diets, while *n* = 3 replicates for 66N and 100 N diets). **B** The apparent digestibility coefficient, ADC, of phosphorous for the reference diet and experimental diets. **C** Digestible grams of phosphorus per kg of feed across the four diets. All error bars reflect standard error relative to the mean. a, b, and c denote similarities and differences between the diets based on the Tukey’s test of multiple comparison

Replacing, or partially substituting, FM with *Nannochloropsis* sp. QH25 decreases the total P in the diet with the total P in the 100N diet (12.7 g P/kg feed) being significantly lower (*p* < 0.001) than the reference diet (18.2 g P/kg feed) (Fig. 2A). The ADC of P% between the reference (73%) and the 100N diet (71%) were similar (*p* > 0.05) (Fig. 2B). All experimental diets had a similar ADC of P% when compared to the reference diet (*p* > 0.05). While the ADC of P% of 33N (70%) was statistically different from the 66N (71%) and 100N experimental diets (*p* > 0.05), the 66N and 100N had a similar ADC of P% relative to one another.

Digestible P was highest in the reference diet and lowest in the 100N diet (13.29 and 8.99 g P/kg diet, respectively) (Fig. 2C). The reference diet and the 33N diets (12.47 g P/kg diet) were similar to each other (*p* > 0.05). Similarly, the 66N and the 100N were statistically similar to each other (*p* > 0.05). However, the reference diet and the 33N were statistically different from the 66N and 100N diets (*p* < 0.001) (Fig. 2C).

Furthermore, the ADC of P of the reference diet and 100N diet were similar (73 and 71%, respectively), indicating that the FM replacing microalgal co-product diet and the reference diet offer comparable amounts of bioavailable P to other species such as tilapia (Gamble et al. 2021). While the total digestible P was highest in the reference diet, this was expected due to the higher initial total P. Larger fish, such as rainbow trout, require less P in their diets for metabolic functions (Sarker et al. 2011), lower levels of total P, and high retention rates, such as the results for the 100N diet, are ideal for fish feeds.

**Fig. 3:**
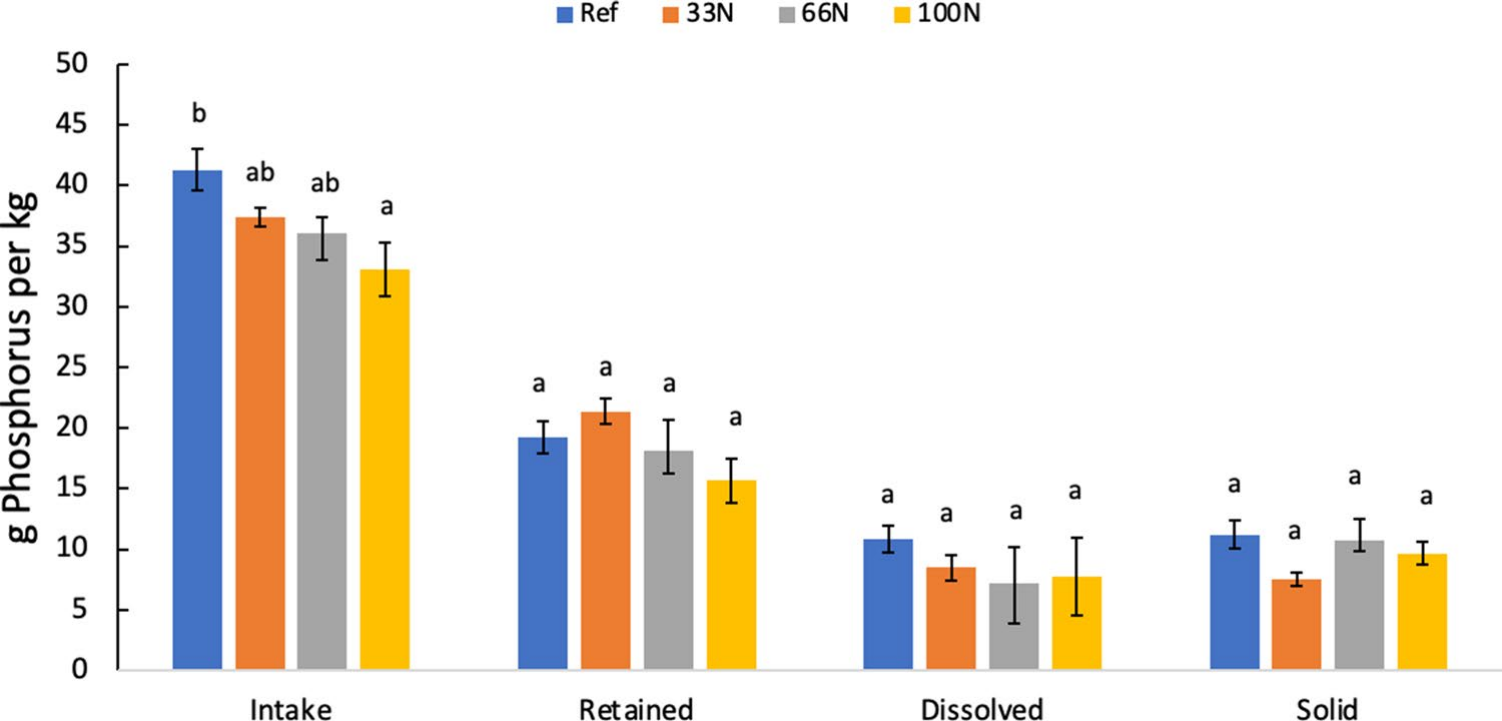
Average (*n* = 4) phosphorus intake, retention, dissolve, and solid waste of *Oncorhynchus mykiss* by the end of the final sampling period. Large rainbow trout were fed a reference (Ref) or experimental diet containing *Nannochloropsis* sp. QH25 that replaced 33N, 66N, or 100N of FM. Error bars reflect the standard error of the mean from the 90-day trial period. a, b, and c denote similarities and differences between the diets based on the Tukey’s test of multiple comparison

P intake was higher in the reference diet when compared to the 100N experimental diet (*p* > 0.05); however, there was no difference in retention between the two diets (*p* > 0.05) (Fig. 3). Rainbow trout that consumed the reference diet had the highest average intake of P per kg of feed (41 g P/kg). However, the P intake of the 33N and 66N diets were similar to each other and all dietary treatments (*p* > 0.05). P retention was higher in the reference diet and lowest in the 100N replacement diet; however, this difference was not significant (*p* > 0.05). P retention was found to be similar across all diets (*p* > 0.05) even though P retention in the 33N diet was larger on average (21 g P/kg).

The reduction of P in aquafeed through replacement FM with *Nannochloropsis* sp. QH25 yielded a lower intake of g of P per kg feed; however, P retention among the experimental diet and all reference diets were similar. Retention of P in aquafeeds through diet manipulation leads to reduced environmental impacts, namely, the eutrophication of freshwater (Sarker et al. 2011; Klinger and Naylor 2012; Gamble et al. 2021; Dalsgaard et al. 2023). While the results were not significant, the 33N diet had the highest P retention, suggesting that the partial substitution of FM using *Nannochloropsis* sp. QH25 could lead to lower eutrophication. The lack of significant difference in retention between the reference diet and experimental diets indicates that P requirements for all groups were met (Sarker 2014).

P loading did not vary significantly between treatments (*p* > 0.05) (Fig. 3). While the average dissolved P loading for the reference diet (10.84 g P/kg feed) was higher than other diets (8.50, 7.15, and 7.73 g P/kg for 33N, 66N, and 100N, respectively), this difference is not significant (*p* > 0.05). Furthermore, the solid P loading was not significantly different between groups (*p* > 0.05). While, on average, the 33N diet has a lower solid P loading (7.53 g P/kg), this difference was not significant (*p* > 0.05) even though solid P loading of other diets (11.21, 10.78, and 9.68 g P per kg for reference, 66N, and 100N, respectively) was larger.

Our results also indicated that *Nannochloropsis* sp. QH25 reduced P loading compared to diets formulated with FM. Many commercial diets rely on terrestrial crop ingredients as replacements for FM; however, many of these ingredients are indigestible to fish (Baruah et al. 2004; Sarker et al. 2020a; Gamble et al. 2021; Dalsgaard et al. 2023) leading to large sums of P loading. Although the results were not significant, the dissolved P of the 100N diet was lower than the reference diet further supporting the usage of *Nannochloropsis* sp. QH25 as a replacement for FM is retained well by the fish. Similarly, though results were not significant, all experimental diets had lower solid P when compared to the reference with the 33N diet having the lowest solid P input of all the diets. The lower levels of solid P indicate that the experimental diets have equal or greater amounts of bioavailable P compared to the reference diet and nutritional needs of the rainbow trout were met (Gamble et al. 2021). The results from this study are consistent with other studies (Schneider et al. 2004; Gamble et al. 2021) in that solid P loading and retention among the reference diet was among the highest values and percentages. Furthermore, this study highlights the importance of considering aquafeeds that yield adequate growth, improve P budget, and tradeoffs of different environmental impacts.

### N digestibility and loading

**Fig. 4:**
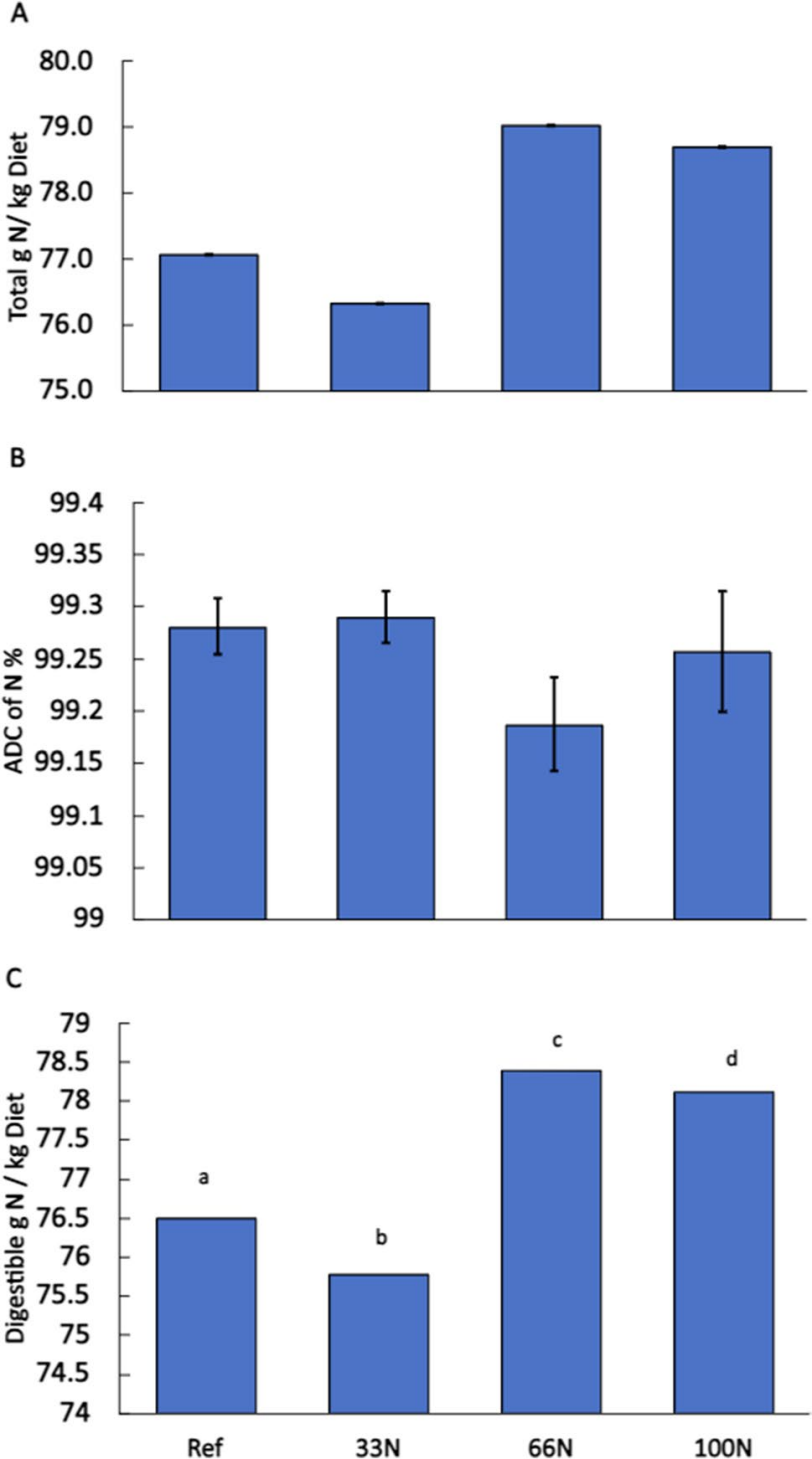
**A** Average quantity of nitrogen in reference and experimental diets. Experimental diets contained *Nannochloropsis* sp. QH25 that replaced 33N, 66N, or 100N of FM in the feeds (*n* = 4 replicates for reference and 33N diets, while *n* = 3 replicates for 66N and 100 N diets). **B** The apparent digestibility coefficient, ADC, of nitrogen for the reference diet and experimental diets. **C** Digestible grams of nitrogen per kilogram of feed across the four diets. All error bars reflect standard error relative to the mean. a, b, c, and d denote significant differences between the diets based on the Tukey’s test of multiple comparison

Replacing, or partially substituting, FM with *Nannochloropsis* sp. QH25 decreases the total N in the 33N diet (77.1 g N/kg feed) and increases the total N in the 66N (76.3 g N/kg feed) and 100N (79.0 g N/kg feed) diets compared to the total N in the reference diet (78.7 g N/kg feed). The total N in the reference and *Nannochloropsis* sp. QH25 diets did not differ significantly (*p* > 0.05) (Fig. 4A). The ADC of P% were similar and not statistically significant (*p* > 0.05) between treatments, ranging between 99.18 and 99.29% (Fig. 4B).

Digestible g N in the diets was significantly higher (*p* < 0.05) in the 100N diet (78.4 g N/kg diet) when compared to the reference diet (76.5 g N/kg diet). All diets were significantly different (*p* < 0.05) in digestible g N (Fig. 4C). The reference diet had a significantly lower (*p* < 0.05) amount of digestible g N/kg diet compared to the *Nannochloropsis* sp. QH25 diets. The 66N diet had the highest amount of digestible g N/kg diet compared to the other diets.

**Fig. 5:**
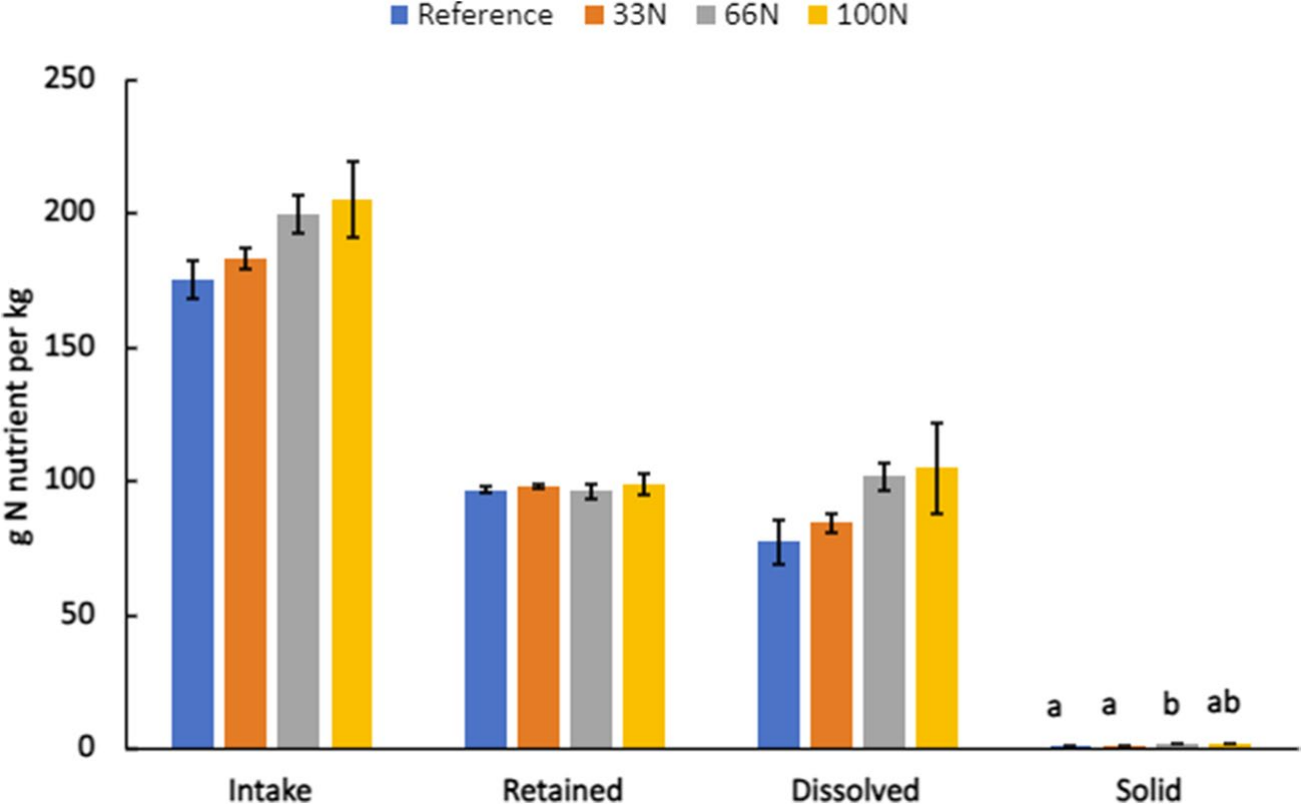
Average (*n* = 4) nitrogen intake, retention, dissolve and solid waste of *Oncorhynchus mykiss* by the end of the final sampling period. Large rainbow trout were fed a reference (Ref) or experimental diet containing *Nannochloropsis* sp*.* QH25 that replaced 33N, 66N, or 100N of FM. Error bars reflect the standard error of the mean from the 90-day trial period. a, b, and c denoted similarities and differences between the diets based on the Tukey’s test of multiple comparison

N intake was highest in the 100N diet (205 g N/kg) but was not significantly different (*p* > 0.05) from the other diets. The reference diet had the lowest average N intake (175 g N/kg). The N intake increased as fish meal was replaced by *Nannochloropsis* sp. QH25, however, did not differ significantly (*p* > 0.05) between all the diets (Fig. 5). N retention was not significantly different across treatments but was highest in the 100N diet (98.8 g N/kg) and lowest in the reference diet (96.0 g N/kg). The solid N loading was significantly higher (*p* < 0.05) in the 66N diet (1.62 g/kg) compared to the other diets (Fig. 5). The amounts for solid N for the other diets were 1.26, 1.30, and 1.51 g P/kg for the reference, 33N, and 100N, respectively.

Although, we found the digestible nitrogen in the *Nannochloropsis* sp. QH25 co-product to be higher than reference diet, the dissolved nitrogen waste emissions did not significantly differ which is similar to our previous findings (Andrade et al. 2024).

### Environmental impact conversion ratios

In addition to looking at P loading, we compared the environmental impact conversion ratios including BRU, GWP, MEP, FEP, water use, and land use of all diets. To achieve a comprehensive sustainability assessment of aquaculture feeds, it is crucially important to consider the environmental impact of aquaculture feeds in terms of greenhouse gas emissions and resource use (Hilborn et al. 2018; MacLeod et al. 2020; Xu et al. 2022; Sarker 2023).

**Fig. 6:**
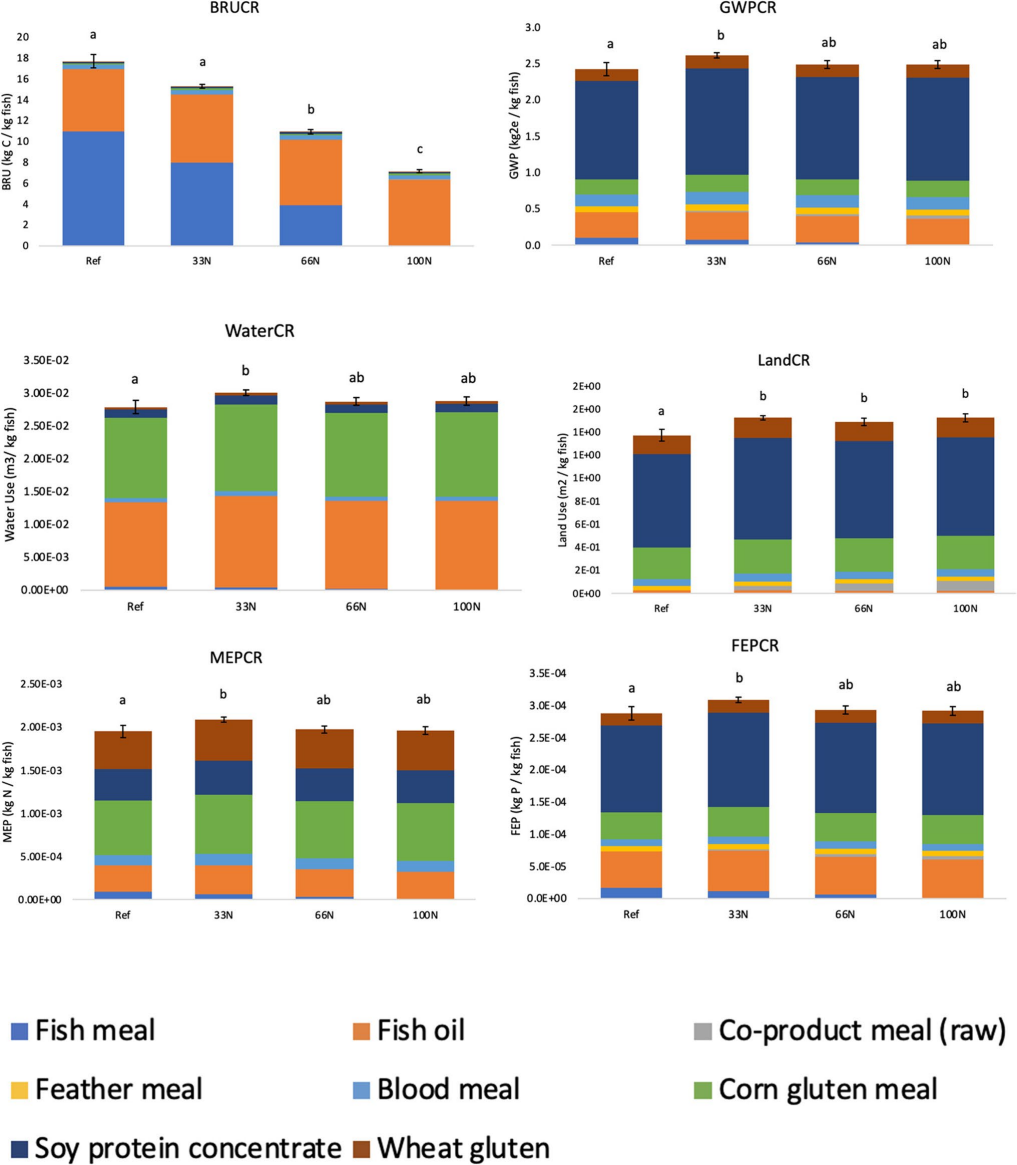
Environmental impact conversion ratio for the reference (Ref), and three experimental diets. The three experimental diets replaced 33% (33N), 66% (66N), and 100% (100N) of the FM with *Nannochloropsis* sp. QH25 co-product meal. The environmental impact conversion ratio is the environmental impact per kg fish and was calculated as the product of the environmental impact and the feed conversion ratio. The environmental impacts included global warming potential (GWP), water use (water), land use (land), marine eutrophication potential (MEP), freshwater eutrophication potential (FEP), and biotic resource use (BRU). Error bars represent standard error of the mean of the FCR. a, b, and c denote similarities and differences between the diets based on the Tukey’s test of multiple comparison

The BRU conversion ratio was significantly higher in the reference diet than in the 100N replacement diet (*p* < 0.01) due to the inclusion of FM (Fig. 6). The environmental impact of the reference diet (17.69 kg C/kg fish) was more than double that of the 100N diet (7.15 kg C/kg fish). All experimental diets had lower BRU conversion ratios; however, only the 66N and 100N diets were significantly lower than the reference diet. Replacing FM can improve or maintain BRU levels at similar levels to a control diet depending on the type of FM replacement (Ghamkhar and Hicks 2021). McKuin et al. (2023) found the replacement of FM with defatted microalgae *Nannochloropsis* sp. had lower BRU and therefore, has the potential to alleviate ocean resource depletion and reduce pressure on marine food webs (Zhang and Kendall 2019). Our results indicate that *Nannochloropsis* sp. QH25 can reduce the BRU impact when implemented in aquafeeds for trout.

There was no significant difference between the GWP conversion ratio of the reference and the 100N diet (2.43 and 2.49 kg CO2e/kg fish respectively) (*p* > 0.05) (Fig. 6). This result has important implications for the sustainability of aquaculture because aquafeed production for salmonids contributes the largest share of greenhouse gasses, GHGs, along the supply chain (MacLeod et al. 2020; Ghamkhar and Hicks 2021). However, there are opportunities to further reduce the GHG emissions of *Nannochloropsis* sp. QH25 which are dominated by urea as a nitrogen source and carbon dioxide as a carbon source in the cultivation process (Ghamkhar and Hicks 2020; McKuin et al. 2023). The GHG emissions of the nutrients for *Nannochloropsis* cultivation could be reduced by the application of circular bioeconomy approaches (Greene and Scott-Buechler 2022). For example, biological carbon sequestration is a method of atmospheric CO_2_ fixation with the production of biomass, which, in turn, can be used as a readily renewable feedstock for the production of biofuels and other valuable products (Sadvakasova et al. 2023). Furthermore, the GHG emissions of the nitrogen source of *Nannochloropsis* sp. cultivation could be reduced by nitrate recycling of industrial or agricultural wastes (Florea et al. 2022).

The water usage conversion ratio of the reference diet (0.028 m^3^/kg fish) was lower than the 100N diet (0.029 m^3^/kg fish) due to the higher feed conversion ratio of the 100N diet. We detected FCR values to be 0.93, 1.01, 0.97, and 0.98 for the reference, 33N, 66N, and 100N diets, respectively. Because higher FCR value indicates that more resources were used to create the feed. However, this difference was not significant (*p* > 0.05). *Nannochloropsis* sp. has negative water use because the freshwater used in the harvesting process was recycled (McKuin et al. 2023). Thus, substituting terrestrial crop ingredients (e.g., soybeans and corn) that have a comparatively higher water demand (Nagappan et al. 2021; Esetlili et al. 2022; Severo dos Santos and Naval 2022) with marine microalgae could improve the overall sustainability of aquafeeds. Despite being a marine microalga, however, *Nannochloropsis* sp. requires a significant amount of freshwater to maintain a constant salinity due to evaporation (Das et al. 2016; Maeda et al. 2018; Al-Jabri et al. 2022). Nevertheless, it may be possible to reduce the freshwater demand to make up for water lost to evaporation and to reduce the need for commercial fertilizers like urea by recycling nutrient-rich wastewater (Van Den Hende et al. 2014; Pugazhendhi et al. 2020). However, the water balance of commercial *Nannochloropsis* sp. production needs to be closely monitored in future studies to better understand the freshwater demands.

Similarly, the land use conversion ratio of the reference diet was significantly lower than all other diets (*p* < 0.05). Of the *Nannochloropsis* sp. QH25 replacement diets, the 100N diets had the highest land usage due to the higher feed conversion ratio; however, the difference was not significant (*p* < 0.05). Replacing the terrestrial crop ingredients (e.g., soybeans and corn) with marine microalgae could reduce the land use of the experimental diets because marine microalgae do not require arable land and have higher biomass yields than terrestrial crops (Benedetti et al. 2018). Furthermore, increasing the use of marine microalgae could alleviate land use changes such as deforestation caused by the use of terrestrial crops (Zhang and Kendall 2019; Nagappan et al. 2021).

We detected no significant (*p* > 0.05) difference in the MEP conversion ratio between the reference diet (0.00195 g N/kg fish) and the 100N diet (0.00197 g N/kg fish). The CAST MEP results showing no significant difference between the experimental 100N and reference has important implications for the sustainability of aquaculture given that excess nitrogen can result in eutrophication (Liu et al. 2019). Additionally, the nitrogen in unconsumed feeds, feces, and dissolved nitrogen are eventually utilized by microbes during the nitrification and denitrification processes wherein nitrous oxide—a powerful greenhouse gas—is generated (Zhou et al. 2021). Thus, improvements in feed utilization could be an effective way to reduce GHG emissions and the eutrophication potential of aquaculture systems. Excess nitrogen is a key contributor to MEP. Our experimental results only considered the role of P in emissions. Future studies should also examine N emissions to get a more representative MEP conversion ratio result.

The FEP conversion ratio of the reference diet (0.000288 kg P/kg fish) was only slightly lower than the 100N diet (0.000292 kg P/kg fish); however, there was no difference between the two diets (*p* > 0.05). Furthermore, the 33N diet had significantly higher FEP conversion ratios (0.00031 kg P/kg fish) when compared to the reference diet (*p* < 0.05). The results from CAST showed that the experimental 100N and reference diets had similar FEP conversion ratios which is in line with our experimental results that did not find significant differences in P retention and P loading. Although the results from CAST were included the experimental feed conversion ratios of this study, the results only included the life cycle impacts of feed production whereas the experimental P results included direct measurements of P emissions into the aquaculture environment. The experimental diets must be manipulated to significantly decrease P loading and increase P retention, therefore decreasing FEP, (Calone et al. 2019; Andrade et al. 2024), while still maintaining optimal growth. Even though P intake was similar, there were no significant differences in the retention, dissolved, and solid waste.

Overall, sustainability improvements can be achieved by improving FCR. One way to achieve this would be by increasing the nutrient quality, feed processing and manufacturing, palatability, and digestibility of the microalgae (Pickova and Mørkøre 2007; Sarker et al. 2013; Nagappan et al. 2021), allowing rainbow trout to consume less feed while maintaining suitable growth rates. Furthermore, it is essential to reduce the reliance on resource-intensive terrestrial crops (e.g. corn and soy-derived ingredients) as these ingredients contribute to high levels of indigestible P, and therefore increased eutrophication impacts, present in experimental diets. The biological efficiency of the diet can also be improved through the manipulation of the ingredients, ensuring that the essential nutrients of the key ingredients in the diet are highly bioavailable for growth.

To achieve this potential, there should be a focus on improving the scale of production, which will ensure the process chain is environmentally sustainable and reduce the cost of production. However, scaling up the continuous production of high-quality microalgal biomass and its downstream processing requires addressing technical, biological, and economic challenges (Hoffman et al. 2017; Acién Fernández et al. 2019). In recent years, there has been a rise in the construction of large-scale cultivation facilities. According to various estimates, these large facilities can produce microalgal biomass at even lower costs (Nagappan et al. 2021). As large-scale microalgae-based industries continue to emerge, the cost of microalgae-based feed is expected to decrease further, making it cost-competitive aquaculture feed.

## Conclusion

Replacing FM with *Nannochloropsis* sp. QH25 co-product did not significantly alter the growth or digestibility of P per kg feed when comparing the 100N and reference diets. Despite the lower total P content in the experimental diet, the P digestibility of the experimental and reference diets was similar, indicating more bioavailable P in the experimental diets. The biotic resource use conversion ratio was significantly lower in the experimental diets when compared to the control. Substituting FM with *Nannochloropsis* sp. QH25 co-product is a step towards aquafeeds that rely less on biotic resources. Despite these successes, using microalgal aquafeed ingredients may have environmental trade-offs as land and water use was significantly higher in the 100N diet compared to the reference diet. GWP, MEP, and FEP were also higher in the 100N diet; however, the differences were not significant. Improving the aquaculture feed production chain is challenging, but there are various ways to enhance the sustainability of aquaculture via formulating feed using alternative feed ingredients. By improving the nutrient efficiency of feed and optimizing microalgae processing and equipment, we can decrease other environmental impacts, such as GWP. It is essential to prioritize technologies that promote energy efficiency through using renewable energy sources to increase sustainability in microalgae production. This will help to reduce the environmental footprint of algae as feed ingredients for sustainable aquaculture.

## Data Availability

All data generated for this study are included in the article.
