# Correction to: Towards cleaner environment: recycling microalgal co-product to reduce emissions and impacts while eliminating fishmeal in rainbow trout feed for sustainable aquaculture

**DOI:** 10.1007/s11356-026-37812-x

**Published:** 2026-05-12

**Authors:** Pallab K. Sarker, Ebenezer Figueroa, Anne R. Kapuscinski, Brandi McKuin, Benjamin V. Schoffstall, Devin Fitzgerald, Connor Greenwood, Kira O’Shelski, Emily Noelle Pasion, Duncan Gwynne, Diego Gonzalez Orcajo, Sofie Andrade, Pablo Nocera

**Affiliations:** 1 Environmental Studies Department, University of California Santa Cruz, Santa Cruz, CA 95060 USA; 2 Earth and Oceanographic Science, Bowdoin College, Brunswick, ME 04011 USA; 3 School of Engineering, University of California Merced, 5200 North Lake Rd, Merced, CA 95343 USA; 4 Department of Microbiology and Environmental Toxicology, University of California Santa Cruz, Santa Cruz, CA 95060 USA

**Correction to: Environmental Science and Pollution Research (2024) 31:46073-46086**

10.1007/s11356-024-34136-6

**First Error: Duration of Experiment**

The experiment duration was 64 days, as correctly stated in the abstract and methods narrative text. However, the wrong number of 90 days was used to produce growth curves and linear regression for Figure 1. Please see below the corrected Figure 1 and caption, presenting the corrected growth curves; and the caption states 64 days and includes corrected linear regression equations and R^2^ values. Note: these corrections did not change the statistical findings of no significant differences, as currently reported in “Results and discussion” under subheading “Growth”.

The wrong “90-day” phrase also needs to be corrected in the captions for Figure 3 and Figure 5.

We also noticed that the first sentence under “Statistical analysis” sub-heading is missing the word “growth” and we provide a corrected sentence below.

Corrected Versions of Figure 1, Figure 1 caption, Figure 3 caption, Figure 5 caption, and sentence under Statistical analysis.
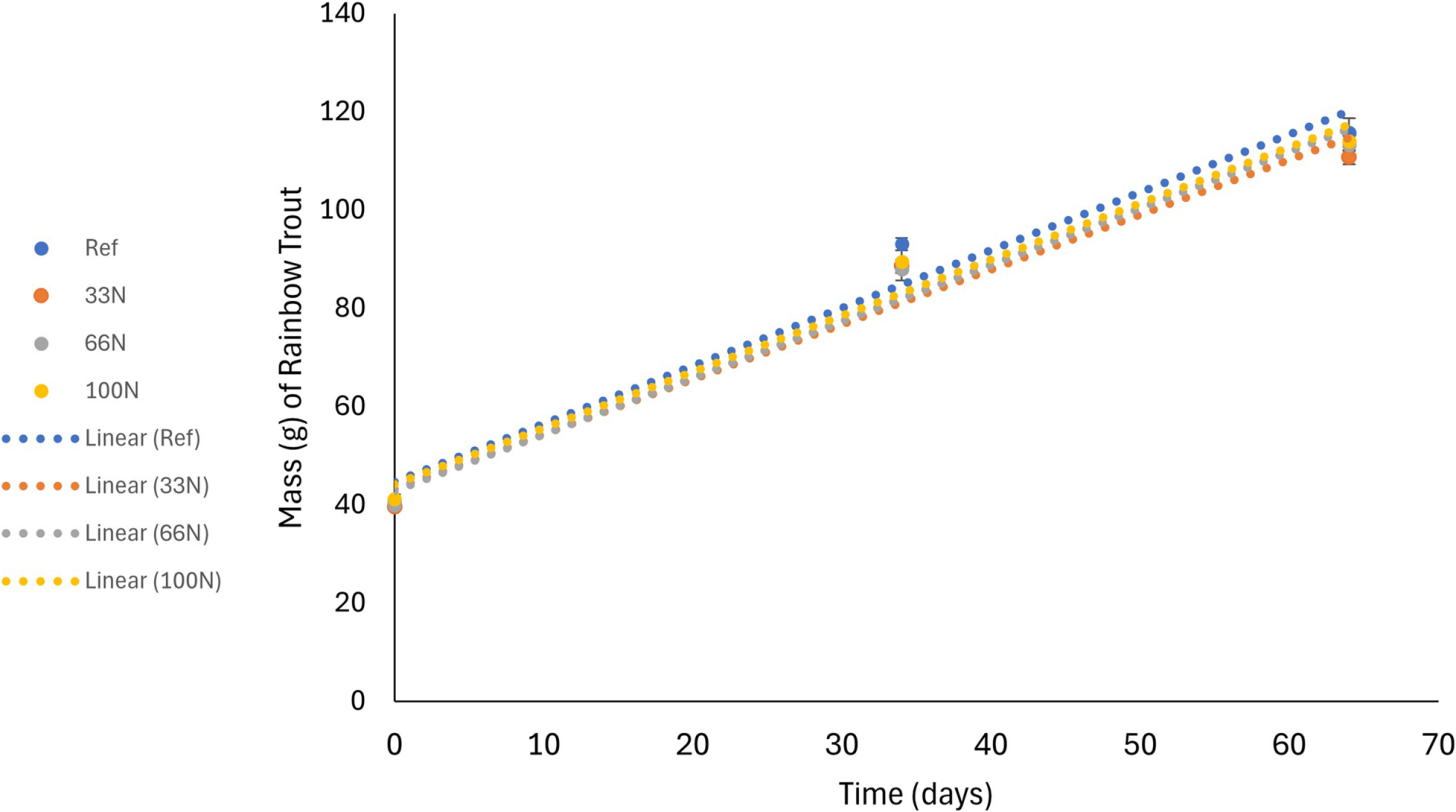


Fig.1 Growth curves for each treatment from day 0 to day 64 for four different diets: no replacement of FM (Ref), replacement of 33% of FM with *Nannochloropsis* sp. QH25*.* (33N), replacement of 66% of FM with *Nannochloropsis* sp. QH25 (66N), and replacement of 100% of FM with *Nannochloropsis* sp. QH25 (100N). Linear regression equations and coefficient of determination (*R*^2^) values: Ref, y = 1.1784x + 44.773 (R^2^ = 0.9652) ; 33N, y=1.1182x + 43.204 (R^2^ = 0.9683); 66N, y = 1.1512x + 42.827 (R^2^ =0.9803); and 100N, y= 1.1438x + 44.204 (R^2^: 0.9775). Error bars show standard error of the mean (*n* = 4 replicates per diet).

Fig 3. Average (*n* = 4) phosphorus intake, retention, dissolve, and solid waste of *Oncorhynchus mykiss* by the end of the final sampling period. Large rainbow trout were fed a reference (Ref) or experimental diet containing *Nannochloropsis* sp. QH25 that replaced 33N, 66N, or 100N of FM. Error bars reflect the standard error of the mean from the 64-day trial period. a, b, and c denote similarities and differences between the diets based on the Tukey’s test of multiple comparison.

Fig 5. Average (*n* = 4) nitrogen intake, retention, dissolve and solid waste of *Oncorhynchus mykiss* by the end of the final sampling period. Large rainbow trout were fed a reference (Ref) or experimental diet containing *Nannochloropsis* sp*.* QH25 that replaced 33N, 66N, or 100N of FM. Error bars reflect the standard error of the mean from the 64-day trial period. a, b, and c denoted similarities and differences between the diets based on the Tukey’s test of multiple comparison

*In the first sentence under “Statistical analysis” sub-heading:*

A one-way ANOVA was run to analyze the variance of growth, P (g/kg), ADC of P, P digested, and P budget (intake, retention, solid, and dissolved) of each diet.

**Second Error: units for Marine Eutrophication Potential (MEP) in sentence under "Environmental impact conversion ratios” subheading**

In the Results section, under the subheading, Environmental impact conversion ratios, sixth paragraph’s first sentence, the wrong unit of“g N” needs to be corrected in two spots; and we note that the MEP panel in Figure 6 gives the correct units of “kg N / kg fish”. The correction is as follows:

We detected no significant (*p* > 0.05) difference in the MEP conversion ratio between the reference diet (0.00195 kg N/kg fish) and the 100N diet (0.00197 kg N/kg fish).

